# Supplementary material for: Type-I nNOS neurons orchestrate cortical neural activity and vasomotion
Source: bioRxiv. 2025 Jan 21:2025.01.21.634042. Preprint. [Version 1] doi: 10.1101/2025.01.21.634042 (PMC11785022; doi:10.1101/2025.01.21.634042)
Supplement: Supplementary file 1 [file NIHPP2025.01.21.634042v1-supplement-1.pdf]

bioRxiv preprint doi: https://doi.org/10.1101/2025.01.21.634042; this version posted January 21, 2025. The copyright holder for this preprint (which was not certified by peer review) is the author/funder, who has granted bioRxiv a license to display the preprint in perpetuity. It is made available under aCC-BY 4.0 International license.

578 **Supplemental Figure 1**

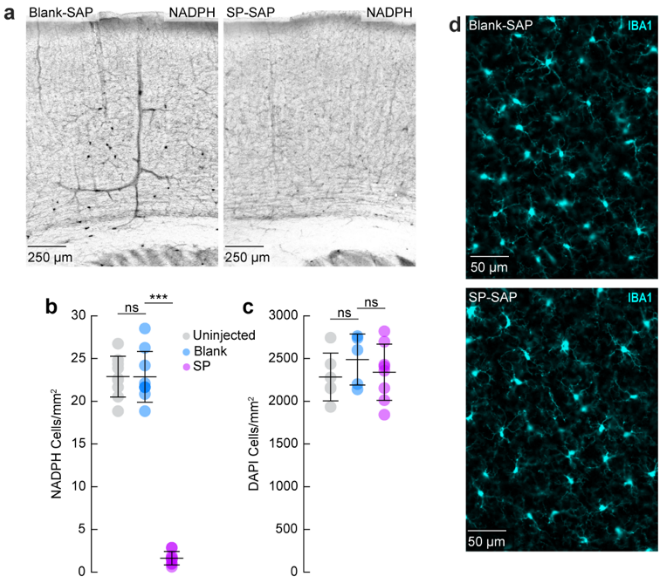

Figure S1. Histological quantification of cortical SP-Sap injections (a) Examples of NADPH diaphorase staining fromBlank-SAP and SP-SAP injected mice. **(b)** SP-SAP injected mice (N = 9, 5M/4F) had significantly lower numbers of type-I nNOS cells than either Blank-SAP (N = 9, 4M/5F) or Uninjected mice (N = 9, 4M/5F) (SP-SAP: 1.6 ± 0.8 neurons/mm^2; Blank-SAP: 22.9 ± 2.4 neurons/mm^2; Uninjected: 22.9 ± 3 neurons/mm^2; Blank-SAP vs. Uninjected: p = 0.98; Blank-SAP vs. SP-SAP: p = 2.23×10^{-13}). **(c)** Counts of DAPI-labeled cell nuclei per square mm from imaged mice. Uninjected (N = 6, 6M) mice had 2284 ± 279 DAPI-labeled cell bodies/mm^2, which was not significantly different than those injected with Blank-SAP (N = 5, 5F, 2488 ± 298, *p* = 0.23, GLME), which in turn was not significantly different than those injected with SP-SAP (N = 8, 4M/4F, 2340 ± 329, *p* = 0.39, GLME. **(d)** Representative image of IBA1 for Blank-SAP (top) and SP-SAP (bottom) taken from **Fig 1c**. Error bars (**b, c**) denote SD. *α < 0.05, **α < 0.01, ***α < 0.001, GLME.

bioRxiv preprint doi: https://doi.org/10.1101/2025.01.21.634042; this version posted January 21, 2025. The copyright holder for this preprint (which was not certified by peer review) is the author/funder, who has granted bioRxiv a license to display the preprint in perpetuity. It is made available under aCC-BY 4.0 International license.

579 **Supplemental Figure 2**

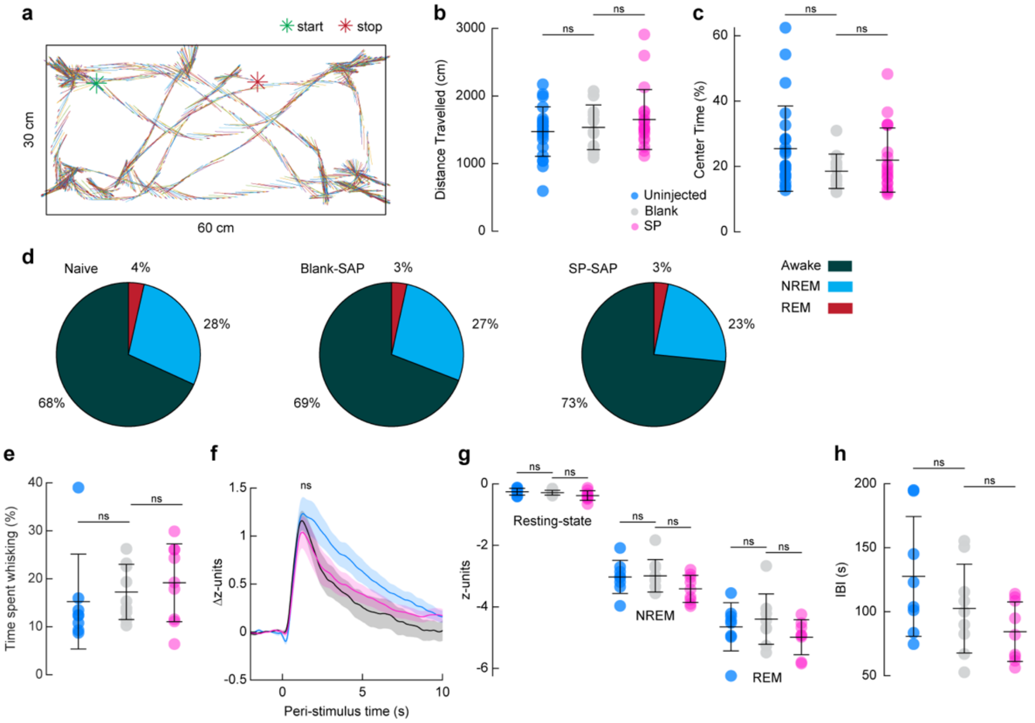

Figure S2. Behavior was unaffected by local ablation of type-I nNOS neurons (a) Example of exploratory behavior during 5 minutes of exploration in a novel environment. **(b)** Total distance traveled during open field exploration. Uninjected mice traveled an average of 1473.4 ± 364.7 cm compared with 1536.6 ± 329.6 cm in Blank-SAP (*p* = 0.61) and 1652.3 ± 442.7 cm in SP-SAP (*p* = 0.43). **(c)** Time spent in the center of the open field. Uninjected mice spent on average 25.5 ± 13.1% of total exploration time in the center compared with 18.5 ± 5.3% in Blank-SAP (*p* = 0.08, GLME) and 21.9 ±9.8% in SP-SAP (*p* = 0.26). **(b, c)** n = 12-23 mice per group. **(d)** Percentage of time classified in each arousal state by sleep scoring. Uninjected mice spent 68.1 ± 13.2% of the time awake compared to 69.1 ± 15.1% in Blank-SAP (*p* = 0.89, ttest) and 73.4 ± 11.6% in SP-SAP (*p* = 0.51, ttest). Uninjected mice spent 28.3 ± 11.1% of the time in NREM sleep compared to 27.4 ± 12.0% in Blank-SAP (*p* = 0.87, ttest) and 23.4 ± 9.7% in SP-SAP (*p* = 0.44, ttest). Uninjected mice spent 3.5 ± 2.9% of the time in REM sleep compared to 3.4 ± 3.5% in Blank-SAP (*p* = 0.96, ttest) and 3.2 ± 2.3% in SP-SAP (*p* = 0.88, ttest). **(e)** Time spent whisking as a percentage of total imaging time. Uninjected mice spent 15.3 ± 9.9% compared with 17.3 ± 5.8% in Blank-SAP (*p* = 0.61, ttest) and 19.2 ± 8.1% in SP-SAP (*p* = 0.75, ttest). **(f)** Change in Pupil diameter in response to vibrissae stimulation. Pupil diameter of uninjected mice increased 1.3 ± 0.5 z-units following vibrissae stimulation compared with 1.2 ± 0.3 z-units in Blank-SAP (*p* = 0.76, ttest) and 1.1 ± 0.5 z-units in SP-SAP (*p* = 0.50, ttest). **(g)** Average pupil diameter across arousal states. Average pupil diameter of Uninjected mice during the resting-state was -0.25 ± 0.1 z-units compared with -0.29 ± 0.1 z-units in Blank-SAP (*p* = 0.45, ttest) and -0.34 ± 0.2 in SP-SAP (*p* = 0.14, ttest). Average pupil diameter of Uninjected mice during periods of NREM sleep was -3 ± 0.5 z-units compared with -3 ± 0.5 z-units in Blank-SAP (*p* = 0.89, ttest) and -3.4 ± 0.4 in SP-SAP (*p* = 0.08, ttest). Average pupil diameter of Uninjected mice during periods of REM sleep was -4.6 ± 0.8 z-units compared with -4.4 ± 0.8 z-units in Blank-SAP (*p* = 0.53, ttest) and -5 ± 0.6 in SP-SAP (*p* = 0.11, ttest) **(h)** Interblink-interval. Uninjected mice had a mean interblink-interval of 127.6 ± 46.8 s compared with 102.5 ± 34.7 s in Blank-SAP (*p* = 0.22, ttest) and 84.5 ± 23.3 s in SP-SAP (*p* = 0.21, ttest). **(d-h)** n = 9 mice per group. Shading **(f)** indicates SEM and error bars **(b, c, e, g, h)** denote SD. *α < 0.05, **α < 0.01, ***α < 0.001.

bioRxiv preprint doi: https://doi.org/10.1101/2025.01.21.634042; this version posted January 21, 2025. The copyright holder for this preprint (which was not certified by peer review) is the author/funder, who has granted bioRxiv a license to display the preprint in perpetuity. It is made available under aCC-BY 4.0 International license.

580 **Supplemental Figure 3**

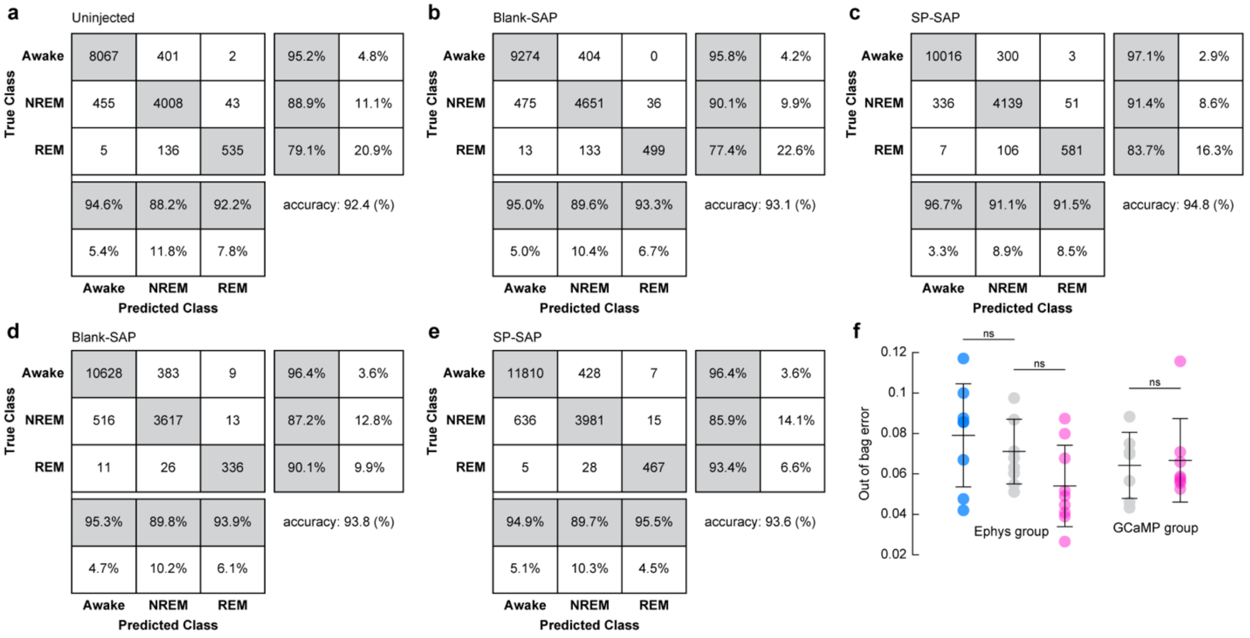

Figure S3. Sleep classification accuracy was unchanged following type-I nNOS ablation (a) Confusion matrix for arousal state classification of Uninjected mice with bilaterally implanted stereotrodes. **(b)** Confusion matrix for arousal state classification of Blank-SAP mice with bilaterally implanted stereotrodes. **(c)** Confusion matrix for arousal state classification of SP-SAP mice with bilaterally implanted stereotrodes. **(a-c)** n = 9 mice per group. **(d)** Confusion matrix for arousal state classification of Blank-SAP mice expressing pan-neuronal GCaMP. **(e)** Confusion matrix for arousal state classification of SP-SAP mice expressing pan-neuronal GCaMP. **(d, e)** n = 6-7 mice per group. **(f)** Out-of-bag error during training of each animal's bootstrapped random forest classification algorithm. For mice with bilateral LFP recordings, Uninjected mice had an average loss of 0.08 ± 0.03 in comparison to 0.07 ± 0.02 in Blank-SAP (p = 0.45, ttest) compared to 0.05 ± 0.02 in SP-SAP (p = 0.064, ttest). Blank-SAP mice with pan-neuronal GCaMP had an average classification loss of 0.06 ± 0.02 compared with 0.07 ± 0.02 in SP-SAP (p = 0.80, ttest). Error bars denote SD. *α < 0.05, **α < 0.01, ***α < 0.001.

bioRxiv preprint doi: https://doi.org/10.1101/2025.01.21.634042; this version posted January 21, 2025. The copyright holder for this preprint (which was not certified by peer review) is the author/funder, who has granted bioRxiv a license to display the preprint in perpetuity. It is made available under aCC-BY 4.0 International license.

581 **Supplemental Figure 4**

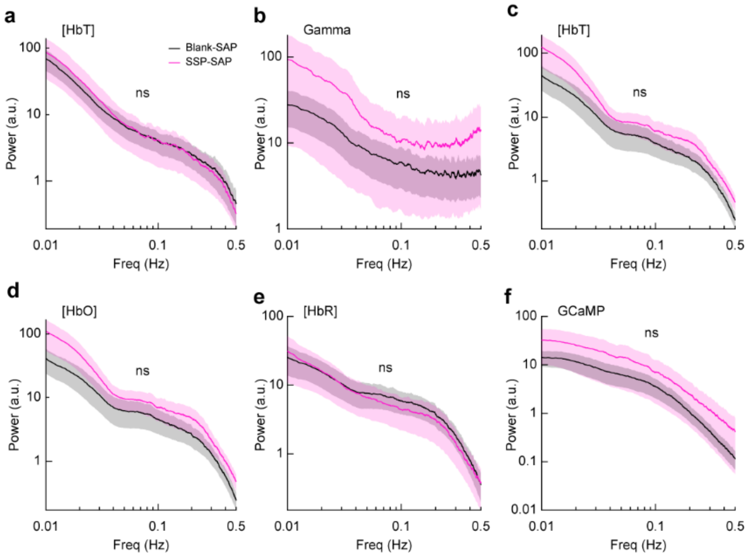

Figure S4. Ablation of Type-I nNOS neurons does not alter hemodynamic or neural power spectra Power spectral density for vascular and hemodynamic signals for the Blank-SAP group (N = 9, 4M/5F for **a, b**; N = 7, 3M/4F for **c-f**) and SP-SAP group (N = 9, 5M/4F for **a, b;** N = 8, 4M/4F for **c-f**) was not significantly different across all measurements of hemodynamic and neural signals. **(a)** Δ[HbT] for electrophysiology animals (p = 0.77, GLME). **(b)** Gamma-band power (second spectra) for animals with electrophysiology (p = 0.19, GLME). **(c)** Δ[HbT] for animals with GCaMP (p = 0.27, GLME). **(d)** Δ[HbT] for animals with GCaMP (p = 0.26, GLME). **(e)** Δ[HbT] for animals with GCaMP (p = 0.68, GLME). **(f)** GCaMP fluorescence (p = 0.44, GLME). Shading represents population averages ± SEM. *α < 0.05, **α < 0.01, ***α < 0.001.

bioRxiv preprint doi: https://doi.org/10.1101/2025.01.21.634042; this version posted January 21, 2025. The copyright holder for this preprint (which was not certified by peer review) is the author/funder, who has granted bioRxiv a license to display the preprint in perpetuity. It is made available under aCC-BY 4.0 International license.

582 **Supplemental Figure 5**

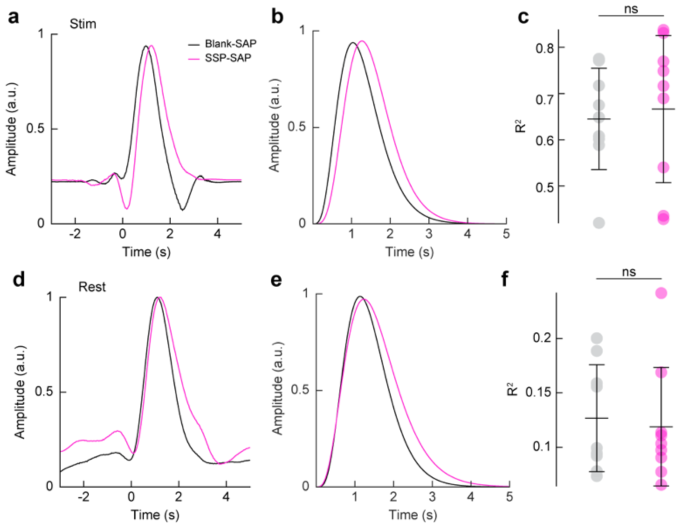

Figure S5. Ablation of Type-I nNOS neurons does not alter the predictive power of the hemodynamic response function (a) Stimulus-evoked hemodynamic response function obtained with deconvolution. **(b)** Stimulus-evoked hemodynamic response function fitted with a gamma distribution function. **(c)** There was no significant difference between the Δ[HbT] predictive R^2 values calculated from impulse-derived HRFs between Blank-SAP (N = 9, 4M/5F) 0.64 ± 0.11 or SP-SAP (N = 9, 5M/4F) 0.67 ± 0.16 following vibrissae stimulation (*p* = 0.746, ttest). **(d)** Resting-state hemodynamic response function based on deconvolution. **(e)** Resting-state hemodynamic response function fitted with a gamma distribution function. **(f)** There was no significant difference between the Δ[HbT] predictive R^2 values calculated from impulse-derived HRFs between Blank-SAP (N = 9, 4M/5F) 0.13 ± 0.0.05 or SP-SAP (N = 9, 5M/4F) 0.12 ± 0.05 during the resting-state (*p* = 0.749, ttest). Error bars denote SD. *α < 0.05, **α < 0.01, ***α < 0.001.

bioRxiv preprint doi: https://doi.org/10.1101/2025.01.21.634042; this version posted January 21, 2025. The copyright holder for this preprint (which was not certified by peer review) is the author/funder, who has granted bioRxiv a license to display the preprint in perpetuity. It is made available under aCC-BY 4.0 International license.

Supplemental Figure 6

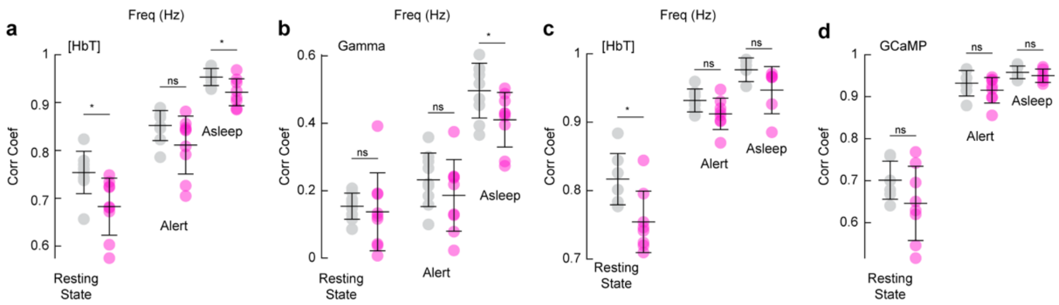

Figure S6. Pearson's correlation coefficients between bilateral hemodynamic and neural signals (a) Correlation coefficient between bilateral hemodynamic signals during the resting-state was 0.75 ± 0.04 in Blank-SAP and 0.68 ± 0.06 in SP-SAP (ttest p = 0.011), during the alert state was 0.85 ± 0.03 with Blank-SAP and 0.81 ± 0.06 in SP-SAP (ttest p = 0.09), and during the asleep state was 0.95 ± 0.02 in Blank-SAP and 0.92 ± 0.03 in SP-SAP (ttest p = 0.01). **(b)** Correlation coefficient between bilateral gamma-band power signals during the resting-state was 0.15 ± 0.04 in Blank-SAP and 0.14 ± 0.12 in SP-SAP (ttest p = 0.69), during the alert state was 0.23 ± 0.08 in Blank-SAP and 0.19 ± 0.11 in SP-SAP (ttest p = 0.31), and during the asleep state was 0.50 ± 0.08 in Blank-SAP and 0.41 ± 0.08 in SP-SAP (ttest p = 0.04). **(c)** Correlation coefficient between bilateral hemodynamic signals (GCaMP7s group) during the resting-state was 0.82 ± 0.04 in Blank-SAP and 0.75 ± 0.04 in SP-SAP (ttest p = 0.01), during the alert state was 0.93 ± 0.02 in Blank-SAP and 0.91 ± 0.02 in SP-SAP (ttest p = 0.08), and during the asleep state was 0.98 ± 0.02 with in-SAP and 0.95 ± 0.03 in SP-SAP (ttest p = 0.07). **(d)** Correlation coefficient between bilateral GCaMP7s signals during the resting-state was 0.70 ± 0.05 in Blank-SAP and 0.65 ± 0.09 in SP-SAP (ttest p = 0.16), during the alert state was 0.93 ± 0.03 with Blank-SAP and 0.92 ± 0.03 in SP-SAP (ttest p = 0.31), and during the asleep state was 0.96 ± 0.02 with Blank-SAP and 0.95 ± 0.02 in SP-SAP (ttest p = 0.37). Error bars denote SD. $\alpha < 0.05$, $**\alpha < 0.01$, $***\alpha < 0.001$.

bioRxiv preprint doi: https://doi.org/10.1101/2025.01.21.634042; this version posted January 21, 2025. The copyright holder for this preprint (which was not certified by peer review) is the author/funder, who has granted bioRxiv a license to display the preprint in perpetuity. It is made available under aCC-BY 4.0 International license.

584 **Supplemental Figure 7**

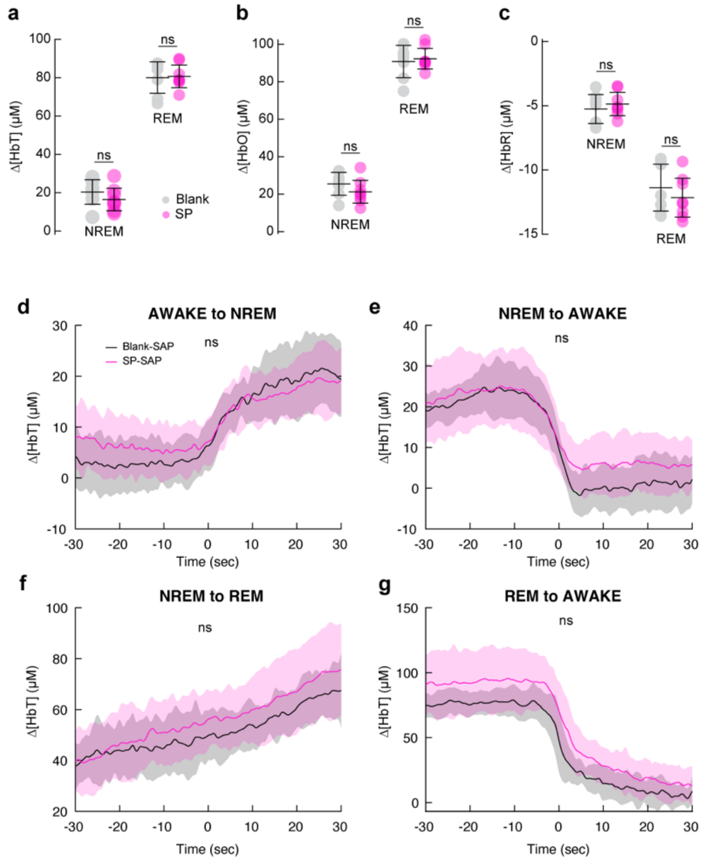

Figure S7. Removal of type-I nNOS neurons did not alter arousal state-related hemodynamic changes (a-c) Average Δ[HbT/O/R] during periods of NREM sleep and REM sleep in mice with pan-neuronal GCaMP (n = 6-7 mice per group). **(a)** NREM Δ[HbT] in Blank-SAP mice was 20.4 ± 6.4 μM compared to 16.5 ± 5.9 μM in SP-SAP (*p* = 0.27, ttest). REM Δ[HbT] in Blank-SAP mice was 80.0 ± 8.2 μM compared to 80.7 ± 5.9 μM in SP-SAP (*p* = 0.87, ttest). **(b)** NREM Δ[HbO] in Blank-SAP mice was 25.5 ± 6.1 μM compared to 21.2 ± 6.1 μM in SP-SAP (*p* = 0.23, ttest). REM Δ[HbO] in Blank-SAP mice was 90.8 ± 8.6 μM compared to 92.3 ± 5.5 μM in SP-SAP (*p* = 0.72, ttest). **(c)** NREM Δ[HbR] in Blank-SAP mice was -5.3 ± 1.1 μM compared to -4.9 ± 0.9 μM in SP-SAP (*p* = 0.50, ttest). REM Δ[HbR] in Blank-SAP mice was -11.4 ± 1.8 μM compared to -12.2 ± 1.5 μM in SP-SAP (*p* = 0.42, ttest). **(d)** Transition from Awake to NREM had a Δ[HbT] of -16.6 ± 6.4 μM in Blank-SAP mice and -11.1 ± 7.0 μM in SP-SAP mice (p < 0.08, GLME). **(e)** Transition from NREM to Awake had a Δ[HbT] of 21.5 ± 7.7 μM in Blank-SAP mice and 17.6 ± 9.5 μM in SP-SAP mice (p < 0.33, GLME). **(f)** Transition from NREM to REM had a Δ[HbT] of -17.6 ± 3.9 μM in Blank-SAP mice and -22.6 ± 7.9 μM in SP-SAP mice (p < 0.09, GLME). **(g)** Transition from REM to Awake had a Δ[HbT] of 67.8 ± 7.3 μM in Blank-SAP mice and 74.0 ± 13.1 μM in SP-SAP mice (p < 0.21, GLME). Error bars denote SD. Shading represents population averages ± SEM.*α < 0.05, **α < 0.01, ***α < 0.001.

bioRxiv preprint doi: https://doi.org/10.1101/2025.01.21.634042; this version posted January 21, 2025. The copyright holder for this preprint (which was not certified by peer review) is the author/funder, who has granted bioRxiv a license to display the preprint in perpetuity. It is made available under aCC-BY 4.0 International license.

585 **Supplemental Figure 8**

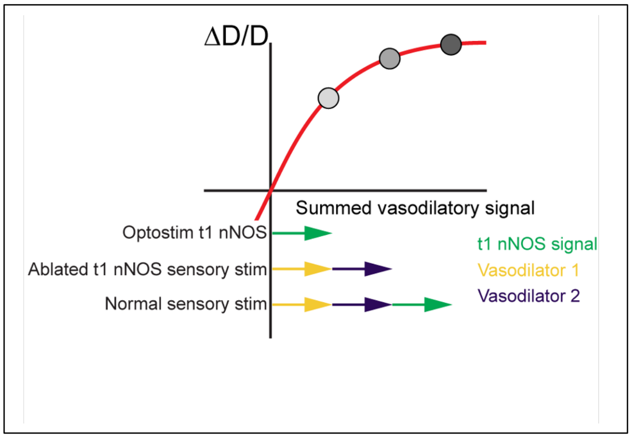

Figure S8. Schematic showing how non-linearity in the dilation response can explain coexistence strong dilation by activation of a pathway, and little change after weakening a pathway. If the diameter is a sublinear function of the sum of vasodilatory inputs, activation of all pathways will cause a dilation that is smaller than the sum of activation of each pathway individually. Loss of one pathway will not cause large changes, even though activation of that pathway in isolation can cause large dilations.
